# Post-stem cell transplant maintenance for pediatric acute leukemias: insights from a Brazilian institution with a Latin American perspective

**DOI:** 10.3389/fonc.2025.1540158

**Published:** 2025-03-05

**Authors:** Carla Nolasco Monteiro Breviglieri, Roseane Vasconcelos Gouveia, Valéria Cortez Ginani, Camila Noronha Santos, Milena Reis Santos de Oliveira, Anna Beatriz Willemes Batalha, Gabriella Sayuri de Alencar, Edna Harumi Goto, Juliana Francielle Marques, Marcia Puato Vieira Pupim, Adriana Seber

**Affiliations:** ^1^ Department of Pediatric Hematology and Hematopoietic Stem Cell Transplantation, Hospital Samaritano Higienópolis, São Paulo, Brazil; ^2^ Support Group for Adolescents and Children with Cancer (GRAACC), Pediatric Oncology Institute, Hematopoietic Stem Cell Transplantation, São Paulo, São Paulo, Brazil

**Keywords:** leukemia, acute lymphoblastic leukemia, acute myelogenous leukemia, hematopoietic stem cell transplantation, maintenance, blinatumomab, hypomethylating agents, pediatric

## Abstract

**Introduction:**

In resource-limited countries, access to advanced therapies like CAR T-cell therapy is often unattainable. Clinical trials face challenges, with pediatric populations frequently excluded or experiencing significant delays. This highlights the need for alternative strategies to address high relapse risks in pediatric acute leukemia post-stem cell transplant.

**Methods:**

This retrospective study included pediatric acute leukemia patients who underwent HSCT between 2014 and 2024. Post-HSCT maintenance therapy became standard practice in 2021, utilizing agents like venetoclax, decitabine, azacitidine, blinatumomab, DLI, and targeted therapies. Primary outcomes were overall survival (OS) and disease-free survival (DFS); secondary outcomes included relapse rate and treatment-related toxicities.

**Results:**

Among 94 patients (64 with ALL, 30 with AML), ALL patients receiving maintenance therapy had an OS of 78% versus 47% without maintenance (p=0.02); DFS was 64% with maintenance and 45% without (p=0.12). In AML patients, maintenance was associated with an OS of 88% compared to 27% without. Relapse rates decreased in maintenance-treated patients, especially among AML patients with pre-transplant MRD positivity. Treatments were generally well-tolerated, with manageable toxicities.

**Discussion:**

Post-HSCT maintenance therapy is feasible in resource-limited settings and may improve survival outcomes. Strategies like hypomethylating agents with venetoclax in T-ALL and post-HSCT blinatumomab in B-ALL show potential benefits. Challenges include drug access and standardizing protocols. Further trials are needed to validate these findings in low- and middle-income countries.

## 1 Introduction

Pediatric acute leukemias are a significant challenge in resource-limited countries, where access to therapies such as CAR T-cells and immunotherapies is restricted due to financial, logistical, and regulatory constraints. While such therapies have transformed treatment outcomes in high-income countries, alternative strategies are essential to address the high risk of relapse following hematopoietic stem cell transplantation (HSCT) in regions with limited resources.

In Brazil, with challenges associated with cost and infrastructure, the experience with CAR T-cell therapy is limited to fewer than 100 patients (1) compared to thousands in the US (2). Tisagenlecleucel (Kymriah^®^), the first commercial CAR T-cell product approved for children and young adults with relapsed or refractory B-cell acute lymphoblastic leukemia (B-ALL), became available in Brazil in 2023. A national survey reported only 15 pediatric patients across seven institutions treated with tisagenlecleucel, with 46% of them achieving durable remission and B-cell aplasia after a median follow-up of 270 days (3). The high cost humpers patient’s access to therapy, so broader funding mechanisms or access to research are urgently needed.

The Brazilian Health Regulatory Agency (Anvisa) has implemented frameworks to ensure safety and monitoring of advanced therapies, facilitating their introduction in the country. The Oswaldo Cruz Foundation (Fiocruz) focuses on reducing costs through the local production of advanced therapies, including CAR T-cell therapy, with the aim of expanding access within the public healthcare system. The University of São Paulo (USP) Ribeirão Preto has pioneered academic CAR T-cell development in the country, with local production at much lower costs. These efforts were recently detailed in a study published in *Bone Marrow Transplantation* journal, demonstrating the feasibility and safety of these initiatives in treating pediatric ALL and yielding promising results (4). Such initiatives highlight the importance of academic research and local production in addressing the economic challenges of advanced therapies in low- and middle-income countries (LMICs).

In areas where CAR-T cells are not widely available to ALL relapsed post-HSCT, it is even more important to prevent disease recurrence. Post-transplant maintenance has been explored to mitigate relapse risk in acute leukemias. While hypomethylating agents (e.g., azacitidine and decitabine) combined with venetoclax are extensively studied in adult acute myeloid leukemia (AML), emerging evidence suggests that these agents may also prevent the relapse of T-cell ALL (T-ALL) (5, 6). A study of four high-risk T-ALL patients receiving post-transplant azacitidine and venetoclax demonstrated durable complete remissions with minimal toxicity, highlighting the potential of these agents for maintenance therapy in this aggressive subtype (5). Similarly, blinatumomab, a bispecific T-cell engager targeting CD19 and CD3, with demonstrated efficacy in relapsed and refractory B-ALL, is being investigated as a maintenance therapy post-HSCT. Recent studies highlight the feasibility and safety in this setting, reducing measurable residual disease (MRD) and prolonging remission (7). Such strategies require careful evaluation, as pediatric-specific data remain scarce, and direct extrapolation from adult studies may not fully account for the unique biology and treatment responses in children. Other post-transplant maintenance approaches in pediatric acute leukemia include the use of tyrosine kinase inhibitors (TKI) for Philadelphia chromosome-positive ALL, FLT3 inhibitors (*e.g.*, sorafenib and gilteritinib) for FLT3-mutated AML, and donor leukocyte infusions (DLI) to prevent the relapse of AML. These strategies, while promising, also require further validation in pediatric populations to optimize their safety and efficacy. Significant disparities in access to all advanced therapies persist, particularly in low- and middle-income countries (LMIC). The financial burden of treatments like CAR T-cells, blinatumomab, venetoclax, FLT-3 inhibitors, TKIs represents a significant barrier, disproportionately affecting families and public healthcare systems in these regions. Addressing these systemic barriers is essential to maximize the impact of emerging therapies and improve outcomes for pediatric acute leukemia globally.

The aim of this study is to report the use and impact of post-HSCT maintenance therapies in pediatric acute leukemias at a Brazilian institution, providing insights into strategies that may be used in similar resource-limited settings, improving outcomes of children transplanted for the treatment of acute leukemias.

## 2 Methods

This retrospective cohort included pediatric patients diagnosed with ALL and AML who underwent HSCT at a single Brazilian institution between January 2014 and December 2024. Patients were younger than 18 years of age at the time of transplant and had high-risk leukemia, defined by positive measurable residual disease (MRD+) or refractory disease before HSCT. Maintenance therapy was implemented as a standard clinical practice in 2021 and the therapeutic strategy was determined based on clinical conditions, access and availability.

Patients with a follow up time greater than six months are grouped as those who received maintenance and those who did not. Few patients transplanted after 2021 did not receive maintenance due to clinical ineligibility, caregiver refusal, or lack of authorization by their health insurance providers. Additionally, a small subset of patients transplanted before 2021 received what was already considered standard maintenance therapies, *i.e.* TKIs for Ph-positive ALL (n=3) and azacitidine for refractory AML (n=2).

Patients started maintenance between 30 and 90 days post-HSCT upon hematologic recovery, usually an absolute neutrophil count (ANC) ≥1.0 × 10⁹/L and a platelet count of ≥50 × 10⁹/L, resolution of acute transplant-related toxicities, such as mucositis and hepatic or renal dysfunction, had no active grade III-IV graft-versus-host disease (GVHD), adequate organ function and absence of infections requiring systemic therapy.

Maintenance strategies varied based on leukemia subtype and clinical characteristics (**Table 1**). In B-cell ALL, the standard regimen included blinatumomab with DLI, except in patients with prior GVHD. Ph-positive ALL patients also received tyrosine kinase inhibitors (TKIs), while venetoclax was added in cases of persistent MRD. Non-responders to blinatumomab in the pre-transplant setting received inotuzumab ozogamicin combined with venetoclax. T-cell ALL patients received hypomethylating agents (decitabine or azacitidine) combined with venetoclax, according to healthcare payer authorization.

**Table 1:** Maintenance strategies.

Leukemia	Maintenance Strategy	Reference
B-cell ALL	Blinatumomab alone or with DLI	Huang J, 2024 (8); Ueda M, 2016 (9)
	Inotuzumab ozogamicin	Metheny LL, 2024 (10)
Philadelphia+ ALL	Tyrosine kinase inhibitors	Brissot E, 2015 (11)
T-cell ALL	Hypomethylating agents with venetoclax	Oran B, 2022 (12); Hassan MA, 2023 (5)
Acute Myeloid Leukemia	Hypomethylating agents alone or combined with venetoclax	Oran B, 2022 (12); Wei Y, 2021 (13); Parks K, 2023 (14); Gao L, 2020 (15); Keruakous AR, 2023 (16)
AML with FLT3 mutation	Sorafenib Gilteritinib	Burchert A, 2020 (17); Levis MJ, 2024 (18)

For patients with AML, maintenance therapy initially included azacitidine in two patients treated before 2021. There on, our standard regimen included a hypomethylating agent combined with venetoclax. From 2023 on, decitabine with G-CSF became the preferred hypomethylating strategy. FLT3 inhibitors were utilized for patients with FLT3-mutated AML. FLT3 inhibitors were used for patients with FLT3-mutated AML: Gilteritinib was administered in 2022 and Sorafenib since 2023.

Venetoclax was a cornerstone of maintenance therapy in both T-ALL and AML, starting with approximately 90 mg/m² (25% of the standard pre-transplant recommended dose) and titrated upwards based on hematological tolerance. Initially, Venetoclax was administered continuously for three weeks, followed by a one-week break in 28-day cycles. However, due to significant hematological toxicity observed in most patients, the administration schedule was shortened to 1–2 week monthly cycles.

### 2.1 Minimal residual disease and chimerism monitoring

Post-transplant disease monitoring was performed to evaluate MRD levels and donor chimerism status. Bone marrow aspirates and donor chimerism assessments were collected monthly during the first year post-HSCT. In the second year, the frequency of monitoring was reduced to once every three months.

MRD levels were primarily assessed using multicolor flow cytometry with an eight-color panel, targeting a sensitivity threshold of 0.01%. For patients with specific molecular markers, such as Ph-positive ALL, BCR/ABL transcript levels were monitored by quantitative PCR (qPCR). FLT3 monitoring was not repeated post-transplant, and next-generation sequencing (NGS) was not employed.

Marrow donor chimerism was evaluated through short tandem repeat (STR). Mixed chimerism identified through these analyses prompted early interventions, including adjustments to immunosuppressive regimens or alteration of maintenance regimen.

### 2.2 Outcome measures

Primary outcomes are overall survival (OS) and disease-free survival (DFS) post-HSCT at the median follow-up for each group. OS was defined as the time from transplantation to death from any cause or the last follow-up. DFS was defined as the time from transplantation to either disease relapse, death from any cause, or the last follow-up, whichever occurred first. Secondary outcomes included relapse rate, toxicities of maintenance therapies, and time to initiation, and duration of maintenance therapy.

### 2.3 Data collection and statistical analysis

Patient data were retrospectively extracted from electronic medical records and included demographics, clinical characteristics, treatment regimens, and outcomes. Adverse events were graded according to the Common Terminology Criteria for Adverse Events (CTCAE, version 5.0). Survival analyses were conducted using Kaplan-Meier methodology, with comparisons between groups performed using the log-rank test. Categorical variables, including relapse rates and toxicity frequencies, were evaluated using Chi-square or Fisher’s exact tests. Statistical significance was defined as a p-value of <0.05, and all analyses were performed using StatPlus software (version 8.0.4.0).

## 3 Results

A total of 94 pediatric patients underwent HSCT in a single institution between 2014 and 2024, including 64 (68%) diagnosed with ALL (10 T-ALL and 54 B-ALL), and 30 (32%) with acute myeloid leukemia (AML).

### 3.1 Patients characteristics

Among patients with ALL, the median age was 8 years (range: 1–16), 74% being male (**Table 2**). Transplants were 50% haploidentical, 41% unrelated, and 9% had matched sibling donors. Stem cell source was 55% peripheral blood, 41% bone marrow, and 4% cord blood. Conditioning regimens were predominantly total body irradiation (TBI)-based (73%). Forty-five percent of patients had measurable disease prior to HSCT, ranging from an MRD of 0.01% to refractory AML with 87% of blasts. The median time to neutrophil engraftment was 18 days (range: 11–30 days).

**Table 2:** Acute lymphoblastic leukemia patients and HSCT characteristics.

Characteristic	Number (%) (n=64)	Maintenance (n=24)	W/o Maintenance (n=40)
Age – median (range)	8 (1–16)	10 (1–18)	7 (1–16)
Female sex	23 (26%)	7 (29%)	16 (40%)
Measurable Residual Disease pre-HSCT			
MRD positive	29 (45%)	12 (50%)	17 (42%)
MRD negative	35 (55%)	12 (50%)	23 (58%)
Second HSCT	8 (13%)	2 (8%)	6 (15%)
HLA-matching/Donor type			
Matched sibling donor	6 (9%)	3 (12%)	3 (7%)
Unrelated donor	26 (41%)	4 (17%)	22 (55%)
Haploidentical donor	32 (50%)	17 (71%)	15 (38%)
Graft source			
Bone Marrow	26 (41%)	9 (37%)	17 (43%)
Peripheral blood	35 (55%)	15 (63%)	20 (50%)
Cord blood	3 (4%)	0	3 (7%)
TBI-based conditioning	47 (73%)	20 (83%)	27 (68%)
Neutrophil engraftment (day post-HSCT), n (range)	18 (11–30)	18 (11–27)	18 (12–30)
Presence of GVHD			
Acute	43 (67%)	16 (67%)	27 (68%)
Chronic	26 (41%)	9 (37%)	17 (43%)

Among patients with AML, the median age was 4 years (range: 0–17), 63% being male. Most transplants were haploidentical (67%), followed by unrelated (27%) and matched sibling donors (6%). Conditioning regimens were predominantly myeloablative and busulfan-based (63%). Over half of the patients (53%) had >5% marrow blasts prior to HSCT. The source of hematopoietic cells was evenly distributed, with 50% of patients receiving bone marrow and 50% receiving peripheral blood stem cells. Three patients (10%) had only MRD-positive disease at transplant (0.7%, 0.4%, and 0.04%). Four patients (13%) had a prior allogeneic HSCT. The median time to neutrophil engraftment was 17 days (**Table 3**).

**Table 3:** Acute myeloid leukemia patients and HSCT characteristics.

Characteristic	Number (%) (n=30)	Maintenance (n=8)	W/o Maintenance (n=22)
Age – median (range)	4 (0–17)	10 (3–17)	4 (0–16)
Female sex	11 (37%)	5 (63%)	6 (27%)
Disease status pre-HSCT			
>5% marrow blasts	16 (53%)	5 (63%)	11 (50%)
MRD positive	3 (10%)	1 (12%)	2 (9%)
MRD negative	11 (37%)	2 (25%)	9 (41%)
Second HSCT	4 (13%)	2 (25%)	2 (9%)
Donor type			
Matched sibling	2 (6%)	1 (12%)	1 (5%)
Unrelated	8 (27%)	0	8 (36%)
Haploidentical	20 (67%)	7 (88%)	13 (59%)
Graft source			
Bone marrow	15 (50%)	4 (50%)	11 (50%)
Peripheral blood	15 (50%)	4 (50%)	11 (50%)
Neutrophil engraftment (day post-HSCT), n (range)	17 (9–30)	16 (15–22)	18 (9–30)
Presence of GVHD			
Acute	21 (70%)	5 (63%)	16 (73%)
Chronic	11 (37%)	5 (63%)	6 (27%)

### 3.2 Patients outcomes

#### 3.2.1 T-ALL outcomes

Among the 10 patients with T-ALL, 6 received maintenance therapy and 4 did not. Maintenance therapy consisted of venetoclax for all patients, with 4 combining it with hypomethylating agents, azacitidine or decitabine. The lack of authorization from health insurance providers was the primary reason for not using maintenance. Venetoclax doses were adjusted based on individual hematological tolerance.

Among the six patients who received maintenance, one relapsed and died due to disease progression, one died from infectious complications and four patients are alive and in remission, with a median follow-up of 23 months. Among the four patients who did not receive maintenance therapy, three were transplanted before 2021 and one had concurrent severe leishmaniasis and thrombotic microangiopathy (TMA). Two of the four patients relapsed and died due to disease progression 62 and 138 days post-HSCT, and one patient died of infectious complications. One of the four patient remains alive and in remission (67-month follow up). Overall, among patients with T-ALL, 16% of those receiving maintenance therapy relapsed, compared to 50% without maintenance.

#### 3.2.2 B-ALL outcomes

Among the 54 patients with B-ALL, maintenance therapy was administered to 18, a third of the patients: blinatumomab (56%), venetoclax (33%), inotuzumab ozogamicin (11%), and dasatinib (28%). One patient with a KRAS mutation received a combination of trametinib and blinatumomab as maintenance (**Table 4**).

**Table 4:** Maintenance strategies.

Drug	B-ALL (n=18)	T-ALL (n=6)
Blinatumomab	10 (56%)	0
Inotuzumab	2 (11%)	0
Venetoclax	6 (33%)	6 (100%)
Dasatinib	5 (28%)	0
Azacitidine/Decitabine	0	4 (67%)

More than one strategy in same patients.

Blinatumomab was administered to 10 patients, 8 of them after haploidentical HSCT, and 9 had received blinatumomab prior to HSCT. Six patients were in their first remission after primary refractory disease, while the remaining four were in second or subsequent remissions. Pre-transplant MRD levels ranged from 0.01% to 0.8% in five patients, while the remaining five patients were MRD-negative prior to HSCT. Conditioning regimens included 1200 cGy total body irradiation (TBI) combined with fludarabine in four patients and etoposide in six patients. Neutrophil engraftment occurred at a median of 18 days post-HSCT (range: 12–22), with blinatumomab initiated at a median of 26 days post-HSCT (range: 22–323). Patients received a median of five cycles of blinatumomab (range: 1–6 cycles), with a 2 week break between cycles (range: 13–44 days).

At the initiation of blinatumomab, seven patients remained on cyclosporine, and two were tapering steroids. Concurrent therapies consisted of dasatinib for a Ph-positive ALL, venetoclax for another, and trametinib for a patient with KRAS-mutated leukemia. To enhance T-cell recovery, five patients without GVHD received concurrent DLI, with a median of two doses per patient and a median CD3+ cell dose of 1.7 × 10⁶/kg (range: 1 × 10⁵–5 × 10⁶/kg). Median lymphocyte counts at the start of the first blinatumomab cycle were 480 CD3/μL (range: 80–2,450).

Adverse events during blinatumomab therapy were generally mild and included hematologic toxicity, viral reactivations (e.g., herpesviruses, CMV, BKV, COVID-19), and fever in four patients. None of them had cytokine release syndrome or severe infections. Infusions were paused for less than 24 hours in patients with fever, before resuming at a slower rate. One patient with Down’s syndrome and positive post-HSCT MRD had a seizure; blinatumomab was paused and restarted at a lower dose with no recurrence of any neurotoxicity. All patients received immunoglobulin replacement during treatment. Seven patients developed acute GVHD, maximal MAGIC grade II, and four had mild chronic GVHD. At a median follow-up of 35 months (range: 3–45 months), seven of the 10 high-risk B-ALL patients treated with blinatumomab remained in continuous remission post-HSCT. An additional patient achieved remission following CAR-T therapy having relapsed after the blinatumomab maintenance (**Table 5**).

**Table 5:** Patients receiving Blinatumomab maintenance post-HSCT.

HSCT	Blina (cycles)	DLI	GVHD	Follow-up (months)	Outcome
Haplo	6	3	Yes	45	Alive - remission
UD	5	No	Yes	42	Alive - remission
MSD	3	No	Yes	38	Alive, remission (after relapse and CAR-T)
Haplo	5	1	No	32	Alive - remission
Haplo	5	2	Yes	44	Alive - remission
Haplo	2	No	Yes	14	Alive - remission
Haplo	5	No	No	9	Alive - remission
Haplo	5	1	No	9	Alive - remission
Haplo	1	1	Yes	3	Dead - remission
Haplo	2	No	Yes	12	Dead - PD

HSCT, allogeneic stem cell transplantation; GVHD, graft versus host disease; Blina, blinatumomab; Haplo, haploidentical; UD, unrelated donor; MSD, matched sibling donor; DLI, donor lymphocyte infusion; PD, progressive disease.

Among the 18 patients with B-ALL receiving maintenance therapy, five relapsed (27%) after a median of 273 days (range: 137–535 days). Two of the 10 patients who received blinatumomab relapsed (20%). In comparison, 36 patients did not receive maintenance therapy and 11 relapsed (31%) after a median of 105 days (range: 63–615 days).

Adverse events associated with maintenance therapy were predominantly mild, including hematologic toxicity and manageable acute or chronic GVHD, with no mortality attributed to infectious complications. Poor graft function or graft failure were observed in either patient group.

**Figure 1:**
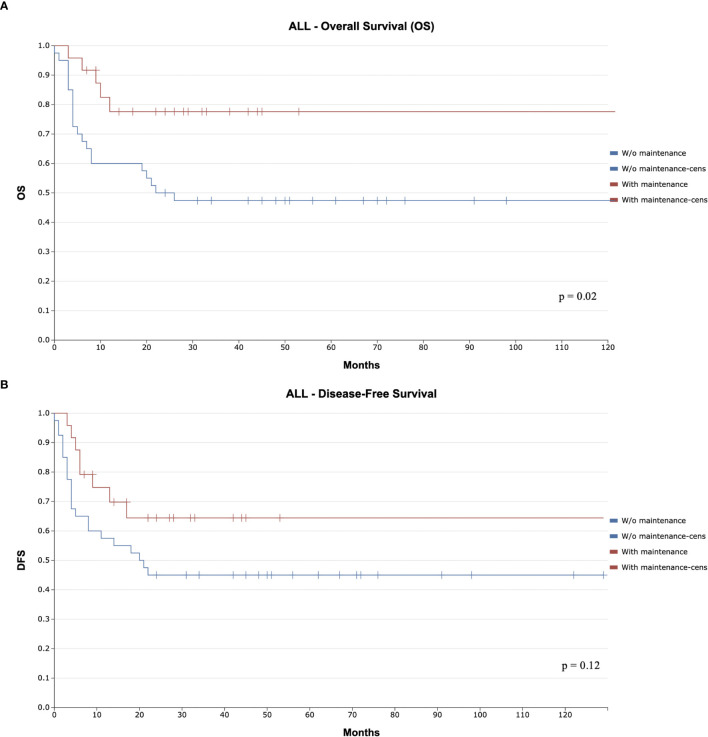
Kaplan-Meier Curve for Overall Survival (OS) and Disease Free Survival (DFS) Comparing Maintenance and Non-Maintenance Groups in Acute Lymphoblastic Leukemia **(A)**. Overall Survival **(B)**. Disease Free Survival.

At a median follow-up of 29 months (7-122 months) for patients with ALL receiving maintenance and 61 months (24-129 months) for those without maintenance, overall survival (OS) was 78% with maintenance and 47% without maintenance (p=0.02; **Figure 1A**). Notably, all deaths without maintenance occurred after the first year of follow-up, with one attributed to disease progression and four to infectious complications. Disease-free survival (DFS) at the median follow-up was 64% with maintenance and 45% without maintenance (p=0.12; **Figure 1B**).

#### 3.2.3 AML outcomes

Among the 30 patients with AML, maintenance therapy was administered to 8 patients (27%), while 22 patients (73%) did not receive maintenance. Maintenance regimens included hypomethylating agents (63%), combined with venetoclax in five of the patients. Two patients with FLT3 mutations received targeted inhibitors: one received gilteritinib in 2022, and another received sorafenib in 2023. Maintenance therapy was initiated at a median of 59 days post-HSCT (range: 32–189 days), with a median duration of 5 months (range: 1–18 months).

The incidence of acute GVHD (grades I–IV) was 70% (21/30) overall, with a higher proportion observed in patients without maintenance group (73%, 16/22) compared to those with maintenance (63%, 5/8). Chronic GVHD was observed in 37% (11/30) of the patients, 63% (5/8) with maintenance and 27% (6/22) without maintenance. Despite this higher incidence of chronic GVHD, no cases exceeded mild-to-moderate severity, and all were managed effectively with standard immunosuppressive regimens.

Among the 11 patients who were MRD-negative at the time of HSCT, 9 did not receive maintenance therapy. With a median follow-up of 4 years, 3/9 relapsed (33%) and subsequently died due to disease progression, 3/9 died of infectious complications, resulting in a total mortality of 67% in this patient group. The median time to relapse without maintenance was 75 days (range: 17–213 days). The two MRD-negative patients who received maintenance remain in remission 2 and 5 years post-HSCT.

In contrast to MRD-negative patients, those with active disease or MRD positivity prior to HSCT (N=19) had distinctly different outcomes. Among the 19 patients in this subgroup, 13 did not receive maintenance therapy and 6 relapsed (46%), with a median time to relapse of 90 days (range: 26–1097 days). Five of these patients died due to disease progression. The patient who experienced the latest relapse remains alive 16 months post-second allogeneic transplant. Of these 13 patients with measurable disease at HSCT but without maintenance, 10 have died: 5 due to disease progression, 1 from late veno-occlusive disease, 1 from severe cardiac failure, and 3 from infectious complications. Among the 6 patients who received maintenance therapy, one child relapsed (17%) 63 days post-HSCT and subsequently died. The remaining five are alive and in remission, with a median follow-up time of 32 months (range: 16–76 months).

Overall, among the 30 AML patients, 10 relapsed (33%) after a median of 77 days (range: 17–1097 days). Specifically, among the 8 patients receiving maintenance therapy, one relapsed (17%) after 63 days, while among the 22 patients who did not receive maintenance, 9 relapsed (41%) after a median of 79 days (range: 17–1097 days).

**Figure 2:**
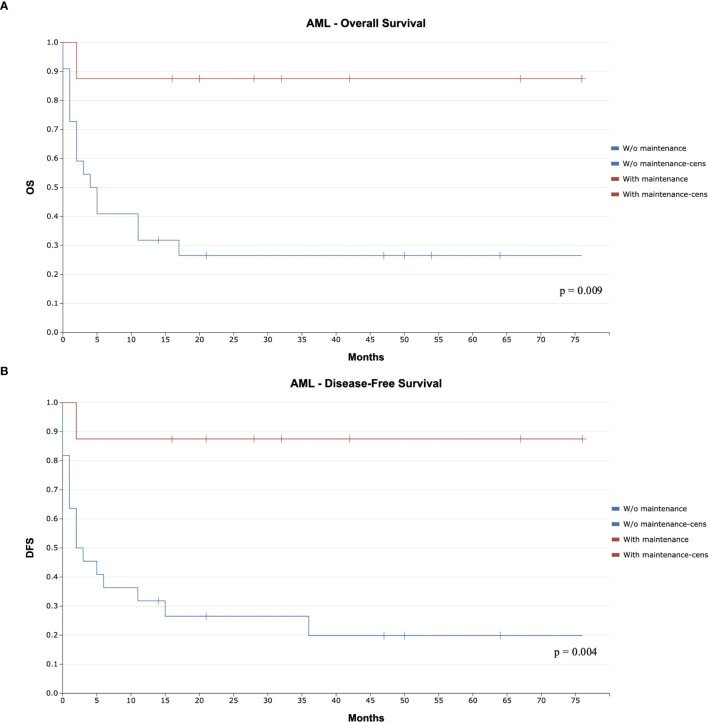
Kaplan-Meier Curve for Overall Survival (OS) and Disease Free Survival (DFS) Comparing Maintenance and Non-Maintenance Groups in Acute Myeloid Leukemia **(A)**. Overall Survival **(B)**. Disease Free Survival. The figures mentioned in the document are included for reference.

At the median follow-up times of 32 months (16-76 months) for patients receiving maintenance therapy and 49 months (14-80 months) for those without, overall survival (OS) was 88% versus 27%, respectively (p=0.009; **Figure 2A**). Similarly, disease-free survival (DFS) was 88% with maintenance, compared to 20% without maintenance (p=0.004; **Figure 2B**).

No poor graft function or graft failure were observed in either group, and adverse events associated with maintenance therapy were predominantly mild, including manageable hematological toxicity and GVHD. Additionally, no deaths were attributed to infectious causes among patients receiving maintenance therapy.

## 4 Discussion

This study demonstrates the feasibility and outcomes of post-transplant maintenance therapies for pediatric acute leukemias in a resource-limited setting, highlighting their potential to improve overall survival (OS) and disease-free survival (DFS). While maintenance therapies are relatively well-established in specific contexts, such as Ph-positive ALL and FLT3-mutated AML, their application in pediatric populations remains underexplored. Our findings contribute to the growing body of evidence suggesting that maintenance therapy after HSCT is both viable and beneficial, even in challenging healthcare environments.

The results show that maintenance therapy was associated with significantly improved outcomes in both ALL and AML. Among ALL patients, OS reached 78% with maintenance, compared with 47% without it, while patients with AML receiving maintenance achieved an OS of 88% versus 27% among those without it. These findings and prior adult studies indicate that maintenance may reduce relapse rates and prolong survival, particularly in high-risk AML populations (19, 20). In our cohort of AML patients, the benefits were most pronounced in those with active disease or MRD positivity at the time of transplant, where maintenance therapy reduced the relapse rate from 46% to 17%.

Notably, relapses in patients with ALL without maintenance therapy occurred early, with a median time to relapse of 105 days without maintenance and 273 days with maintenance. This delay in relapses of ALL in patients receiving maintenance therapy may have clinical implications, since later relapses, although still challenging to treat, are generally more amenable to salvage therapies compared to early relapses. Conversely, in AML patients, the available data are insufficient to determine whether maintenance therapy delays relapses. Only one patient with maintenance relapsed, limiting comparisons with patients without maintenance. However, maintenance therapy in our children with AML reduced relapse rate from 41% to 17%. These findings underscore the importance of maintenance therapy in reducing relapse rates across leukemia subtypes, even though its impact on relapse timing may vary depending on the disease context and underlying biology.

In ALL, the dual benefits of maintenance therapy—reducing relapse rates and delaying eventual relapses—are evident, particularly for high-risk patients where alternative options may be limited.

The efficacy of hypomethylating agents combined with venetoclax in T-cell ALL (T-ALL) is particularly noteworthy. T-ALL remains one of the most difficult leukemias to treat after relapse, due to the lack of CAR T-cell therapies for T-lineage leukemias and limited access to other effective drugs, such as nelarabine, in resource-constrained settings. In our study, the combination of hypomethylating agents with venetoclax achieved durable remissions in this high-risk population, representing a critical therapeutic alternative in cases where options are otherwise limited. These findings are significant, given that relapsed T-ALL is typically associated with poor prognoses, and the results of our cohort suggest a feasible and effective strategy for these patients. The success of this combination underscores the need for further investigation in prospective pediatric trials to validate and optimize this approach.

Blinatumomab also demonstrated efficacy as maintenance for B-ALL. Patients receiving blinatumomab post-HSCT experienced a 20% relapse rate compared to 31% without maintenance, despite much worse prognosis as reflected by pre-HSCT positive MRD. The administration of blinatumomab post-HSCT remains largely unexplored in pediatric cohorts, making our findings very interesting to be further explored. Furthermore, the recent development of subcutaneous formulations of blinatumomab, as highlighted in recent studies (21), could simplify its administration, reduce hospitalization requirements, and improve accessibility for post-transplant patients in resource-limited settings. This innovation could have a particular impact in reducing logistical and financial burden, while maintaining efficacy, further solidifying its role as a cornerstone of maintenance therapy in B-ALL.

However, this study also underscores the challenges of implementing maintenance therapy in pediatric settings, particularly in LMICs. Variability in maintenance regimens also reflects the real-world constraints of resource-limited settings. In B-cell ALL (B-ALL), maintenance strategies ranged from single-agent blinatumomab to combinations with venetoclax or targeted therapies such as trametinib and dasatinib. Furthermore, delays in therapy initiation, often caused by health insurance authorization processes, likely impacted outcomes and underscore the systemic barriers to implementing timely maintenance. Despite these challenges, the observed reduction in relapse rates and prolongation of survival emphasize the potential of maintenance therapy to mitigate high relapse risks in this population.

The cost-effectiveness of maintenance therapy is another important consideration, particularly in LMICs. Relapses are associated with significant financial and emotional burden due to hospitalization, salvage therapies, and, in some cases, the need for CAR T-cell therapy or second transplants. Our findings suggest that maintenance therapy may improve outcomes and ultimately reduce overall healthcare costs by preventing relapses.

In patients with active AML or positive MRD pre-HSCT, the reduction in relapse rates with maintenance therapy highlights its potential to avoid costly interventions, offering a pragmatic strategy in resource-constrained environments.

Despite these promising results, the study highlights important barriers to access in specific cases. While nearly three-quarters of AML patients and two-thirds of ALL patients did not receive maintenance therapy, the majority of these cases occurred prior to 2021, before maintenance therapy was routinely implemented as part of clinical practice. In some patients, after 2021, therapy initiation was delayed by weeks or months due to logistical challenges, and in others, patients received incomplete regimens due to limitations in drug availability or insurance coverage. These disparities likely influenced outcomes and underscore the urgent need for systemic reforms to facilitate equitable access to maintenance therapies.

Adverse events associated with maintenance therapies were generally manageable, with no cases of poor graft function or graft failure in the maintenance group and few deaths attributed to infectious causes in both groups. However, chronic GVHD was more common with maintenance, affecting 63% of the patients, compared with 27% without maintenance, although all mild to moderate and well-controlled with standard immunosuppression. The higher incidence of chronic GVHD with maintenance may reflect enhanced immune reconstitution associated with these therapies, but warrants further investigation of the immunomodulatory effects.

The study has several important limitations: it was not a clinical trial, patients had multiple diagnoses and interventions, data was retrospectively evaluated, follow-up is heterogeneous. While the median follow-up time differed between groups, it is long enough to capture most relapses, which predominantly occur within the first months post-HSCT. The follow-up exceeded the typical time to relapse observed in both AML and ALL, emphasizing the importance of early maintenance initiation to mitigate the risk of relapse when risk is highest.

In conclusion, this study demonstrates the feasibility and potential benefits of post-HSCT maintenance therapy for pediatric acute leukemia in a resource-limited setting. Despite challenges related to access, standardization, and cohort size, maintenance therapy was associated with improved OS and DFS, particularly among high-risk patients. The significant benefits observed with hypomethylating agents combined with venetoclax in T-ALL and the efficacy of post-HSCT blinatumomab in B-ALL highlight the transformative potential of these approaches, particularly in underserved pediatric populations. The availability of subcutaneous blinatumomab may increase access to this therapy, making it an attractive option for post-HSCT care. Our findings still underscore the importance of integrating surveillance and maintenance strategies into the post-transplant care to reduce the risk of relapse, optimize resource utilization, and improve outcomes of children transplanted for the treatment of acute leukemias.

## Data Availability

The original contributions presented in the study are included in the article/supplementary material. Further inquiries can be directed to the corresponding author.
